# Protein Structural Dynamics of Wild-Type and Mutant Homodimeric Hemoglobin Studied by Time-Resolved X-Ray Solution Scattering

**DOI:** 10.3390/ijms19113633

**Published:** 2018-11-18

**Authors:** Cheolhee Yang, Minseo Choi, Jong Goo Kim, Hanui Kim, Srinivasan Muniyappan, Shunsuke Nozawa, Shin-ichi Adachi, Robert Henning, Irina Kosheleva, Hyotcherl Ihee

**Affiliations:** 1 Department of Chemistry and KI for the BioCentury, Korea Advanced Institute of Science and Technology (KAIST), Daejeon 34141, Korea; eangel04@gmail.com (C.Y.); atlady05@gmail.com (M.C.); 8808kjk@gmail.com (J.G.K.); haneihanei92@gmail.com (H.K.); smuniappan@gmail.com (S.M.);; 2 Center for Nanomaterials and Chemical Reactions, Institute of Basic Science (IBS), Daejeon 34141, Korea; 3 Institute of Materials Structure Science, High Energy Accelerator Research Organization (KEK), 1-1 Oho, Tsukuba, Ibaraki 305-0801, Japan; noz@post.kek.jp (S.N.); shinichi.adachi@kek.jp (S.-i.A.);; 4 Department of Materials Structure Science, School of High Energy Accelerator Science, The Graduate University for Advanced Studies, 1-1 Oho, Tsukuba, Ibaraki 305-0801, Japan; 5 Center for Advanced Radiation Sources, The University of Chicago, Chicago, IL 60637, USA; henning@cars.uchicago.edu (R.H.); ikoshelev@cars.uchicago.edu (I.K.);

**Keywords:** homodimeric hemoglobin, time-resolved X-ray solution scattering, allostery, molecular cooperativity, protein dynamics

## Abstract

The quaternary transition between the relaxed (R) and tense (T) states of heme-binding proteins is a textbook example for the allosteric structural transition. Homodimeric hemoglobin (HbI) from *Scapharca inaequivalvis* is a useful model system for investigating the allosteric behavior because of the relatively simple quaternary structure. To understand the cooperative transition of HbI, wild-type and mutants of HbI have been studied by using time-resolved X-ray solution scattering (TRXSS), which is sensitive to the conformational changes. Herein, we review the structural dynamics of HbI investigated by TRXSS and compare the results of TRXSS with those of other techniques.

## 1. Introduction

As a prime example of the allostery, the quaternary structural transition of human tetrameric hemoglobin (Hb) induced by ligand binding and dissociation has been investigated extensively [1,2,3,4,5,6,7]. However, the allosteric structural transition of Hb is still elusive because of the complex kinetics arising from its heteromeric tetramer structure. In this respect, homodimeric hemoglobin (HbI) from *Scapharca inaequivalvis* has served as an excellent model system for investigating the allosteric structural transition between a ligated R state with high ligand affinity and a deoxygenated T state with low ligand affinity [8,9,10,11,12,13,14,15,16,17,18,19,20,21,22,23,24,25,26,27,28,29,30,31,32,33,34,35,36,37,38] due to its simpler dimeric structure. In the HbI, the E and F helices of monomers are located at the interface of homodimer [11,12,39], which is often referred to as EF dimer. This EF dimer structure has also been found in tetrameric hemoglobin from *Caudina arenicola* [40] and erythrocruorin from *Lumbricus terrestris*, which is a megadalton complex including 144 hemoglobin subunits and 36 linker subunits [41]. In this respect, studies on the allostery of HbI would provide a clue for understanding the structural dynamics of the larger and complex hemoglobin. On the other hand, the EF dimer assemblage of HbI is different compared with the assemblage of tetrameric Hb, whose E and F helices are facing the solvent [12]. In this regard, studies on the structural dynamics of HbI would be helpful to better understand how the diverse quaternary structures of hemoglobins are connected with the variety of mechanisms for the allosteric regulation of the ligand binding.

The structural dynamics of HbI has been studied by various time-resolved experimental tools such as time-resolved X-ray crystallography [25,26,27] and transient absorption (TA) spectroscopy [9,13,17,20,22,28,29,30]. Time-resolved X-ray crystallography can provide detailed structures of intermediates with a high atomic resolution, but its application is limited because the large structural changes of proteins are often constrained in the crystal phase. For instance, it has been reported that the quaternary rotation of subunits in HbI in the crystal phase determined by time-resolved X-ray crystallography is not fully completed within 80 μs and the angle of the subunit rotation is only 0.6° instead of 3.3°, which is the rotation angle difference between the static R and T states in the crystal [25,26]. Due to the constraint of the crystal phase, methods for determining the conformation of a protein in solution phase have been spotlighted. For example, Khoshouei et al. showed that the cryo-electron microscopy (cryo-EM) technique with the Volta phase plate can be used for the structure determination of relatively small proteins, such as Hb and myoglobin in solution [42,43]. In the study using cryo-EM, authors determined the structure of Hb with a resolution of 3.2 Å and observed water molecule densities, which are consistent with the positions of conserved water molecules in a crystal structure [42]. Even though there are powerful techniques, such as cryo-EM and nuclear magnetic resonance (NMR) spectroscopy, for determining the static structures of proteins in solution, time-resolved studies in solution with sufficient time resolution are essential for understanding the characteristics of the reaction intermediate. In this respect, TA spectroscopy is an excellent time-resolved technique to investigate the kinetics of proteins in solution by probing the absorption changes with femtosecond time resolution. However, TA spectroscopy is sensitive only to the local conformational changes of the chromophore and its nearby environment and is generally less sensitive to the global structural changes [44,45,46].

In this regard, time-resolved X-ray solution scattering (TRXSS), also known as time-resolved X-ray liquidography (TRXL), is a complementary experimental tool and has been used for investigating the structural dynamics of various protein molecules as well as small molecules [21,24,47,48,49,50,51,52,53,54,55,56,57,58,59,60,61,62,63,64,65,66,67,68,69,70,71,72,73,74,75,76,77,78,79,80,81]. For example, in a picosecond TRXSS study of sperm whale myoglobin, the kinetic model including the biphasic transition from the first to the second intermediates was established and the biphasic kinetics was elucidated as a result of the involvement of two conformational substates of the first intermediate [75]. In a femtosecond TRXSS study of horse myoglobin at an X-ray free-electron laser (XFEL), the time evolution of the radius of gyration and the volume revealed an oscillatory collective motion of the atoms in myoglobin, which was damped with a 6 ps time constant, and the authors postulated the intrinsic ballistic-like nature of protein motion that is usually undetected in ensemble measurements at longer timescales [65]. Additionally, the light-induced structural deformation of the photosynthetic reaction center, which is the direct structural evidence of the protein quake in the solution phase, was captured by using a femtosecond TRXSS study [62]. TRXSS has an even wider application in small molecules. For example, a study of a gold trimer complex, [Au(CN)_2_^−^]_3_, using femtosecond TRXSS demonstrated that the structural changes, such as covalent bond formation, during a chemical reaction in solution can be tracked in real time by using XFEL and an advanced analysis of TRXSS data [64]. As demonstrated in these examples, TRXSS with high temporal and spatial resolutions can provide valuable information on the structural dynamics of proteins and small molecules.

**Figure 1:**
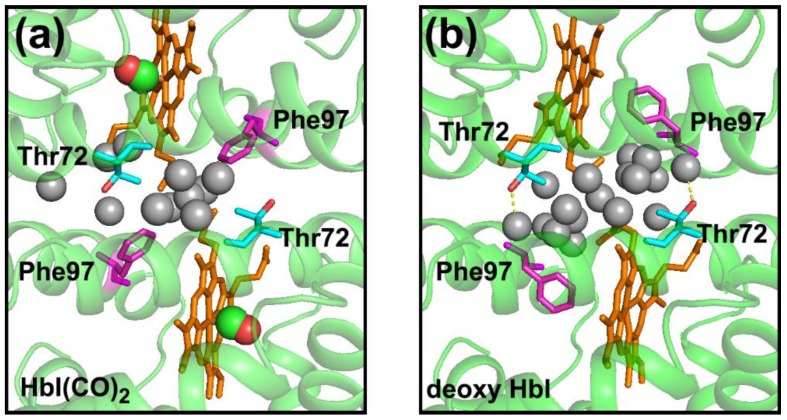
(**a**) The magnified view of the subunit interface of the crystal structure of WT HbI(CO)_2_ (PDB ID: 3sdh) and (**b**) that of deoxy WT HbI (PDB ID: 4sdh). Protein structures are shown in cartoon representations (green). Eleven and seventeen interfacial water molecules for HbI(CO)_2_ and deoxy HbI, respectively, are shown with gray spheres. Two CO ligands are shown with connected red and green spheres. Heme, Thr72, and Phe97 are represented in orange, cyan, and magenta sticks, respectively. In the deoxy HbI, two interfacial water molecules interact with the hydroxyl group of Thr72 (red) via hydrogen bonds (yellow dotted lines).

According to X-ray crystallography studies [11,12], the static structures of the R and T states of wild-type (WT) HbI have the following characteristics. The R and T states of HbI have different angles between two monomer subunits, indicating that the subunits are rotated against each other during the R-T transition. Additionally, the distance between the heme irons of two monomers in the T state is smaller than that of the R state. The number of the interfacial water molecules between the two monomers of the T state is larger than that of the R state (Figure 1 and Table 1) [11,12,13,14,15]. In the case of the crystal structures of WT HbI, deoxy HbI shows 17 well-ordered interfacial water molecules having an average B-factor of 19.6 Å^2^, while HbI ligated with CO ligands (HbI(CO)_2_) shows 11 well-ordered interfacial water molecules having an average B-factor of 24.7 Å^2^ [17]. In a study of HbI using crystallography and resonance Raman spectroscopy, it was suggested that the water cluster at the subunit interface plays a crucial role in the cooperative functioning of HbI [13]. Among the residues related to the water cluster at the subunit interface, two residues of Phe97 (F97) and Thr72 (T72) are of interest. The F97 residue is located under the heme plane and nearby the proximal histidine residue in the T state, whereas the side chain of F97 is flipped out of the space under the heme and exposed to the subunit interface in the R state (Figure 1). The exposed side chain of F97 in the R state displaces three water molecules directly [17]. As a result of the displacement of three water molecules by each F97 residue, the HbI R state loses six of the well-ordered interfacial water molecules compared with the T state (Figure 1 and Table 1). In the case of the F97Y mutant of HbI (F97Y HbI), it was suggested that even the deoxy state of the mutant is locked in the R state instead of the T state since the deoxy state of F97Y HbI has a higher ligand affinity and many of the structural properties such as positions of hemes that does not attain the complete T state position [17]. Since the phenol side chains of two Tyr97 residues remain in the subunit interface in both the ligated and deoxy states, the interfacial water cluster of F97Y HbI is significantly altered in both the ligated and deoxy states compared with that of WT HbI (Table 1) [17]. The side chain of the T72 residue interacts with one of the interfacial water molecules in the deoxy T state (Figure 1b) and the interaction between T72 and the water molecule stabilizes the T state [13,23]. In the ligated R state, instead of the interaction with the water molecule, the methyl group of the side chain of the T72 residue packs against the F97 residue of another subunit [23]. Due to the lack of the hydroxyl side chain of the T72 residue, the deoxy state of the T72V mutant HbI (T72V HbI) has two less interfacial water molecules compared with that of WT HbI, while the ligated states of T72V HbI and WT HbI have the same number of interfacial water molecules (Table 1) [13].

Even though the structures of the two end states [11,12,13,14,15,17,23] and the structural dynamics [9,13,17,20,22,25,26,27,28,29,30] of HbI have been studied extensively, detailed structural dynamics of HbI in the solution phase associated with the allosteric effect had not been understood completely. To understand the role of the subunit interface in the allosteric structural transition, WT, F97Y, and T72V HbI have been studied by using TRXSS [21,24]. As a result of TRXSS studies, the intermediate structures in the solution phase and the kinetic parameters were obtained based on a kinetic model commonly applicable to WT, F97Y, and T72V HbI. Importantly, the TRXSS results revealed that the F97Y and T72V mutations, which perturb the subunit interface of HbI, inhibit the contraction of the heme–heme distance during the R–T transition after photolysis while retaining the subunit rotation similar to WT HbI. In this review, we summarize the structural dynamics of HbI studied by using TRXSS and compare the results of TRXSS with those of other techniques.

## 2. Structural Dynamics of HbI studied by TRXSS

### 2.1. Data Acquisition of TRXSS Data

Detailed descriptions for data collection of TRXSS data are available in previous publications [21,24]. TRXSS data of HbI were collected by using the pump-probe scheme at the NW14A beamline at KEK [21] and 14IDB beamline at the Advanced Photon Source (APS) [21,24]. A typical experimental procedure is described as follows. Sample solutions of HbI(CO)_2_ for WT, F97Y, and T72V HbI were prepared by following a previously reported protocol [82]. A sample solution contained in a capillary was excited by ~35 ps laser pulses of 532 nm wavelength from the top. The X-ray pulse passes through the capillary in the perpendicular geometry. In the case of the standard operating mode (24-bunch mode) of the APS, each X-ray pulse has a temporal width of 100 ps (full width at half maximum). Two-dimensional X-ray scattering patterns were collected with a two-dimensional MarCCD detector at time delays (Δ*t*) in the range from 100 ps to 56.2 ms and converted into one-dimensional scattering curves by azimuthal integration. The time-resolved difference scattering curves, Δ*S*(*q*, *t*) = *S*(*q*, *t*) – S(*q*, –5 μs), were obtained by subtracting the scattering curve at –5 μs from the curves at positive time delays. To remove the contribution from solvent heating, a difference scattering curve at a late time delay was subtracted from the difference scattering curves at all time delays [21].

### 2.2. Structure Refinement Used for the Analysis of TRXSS Data

**Figure 2:**
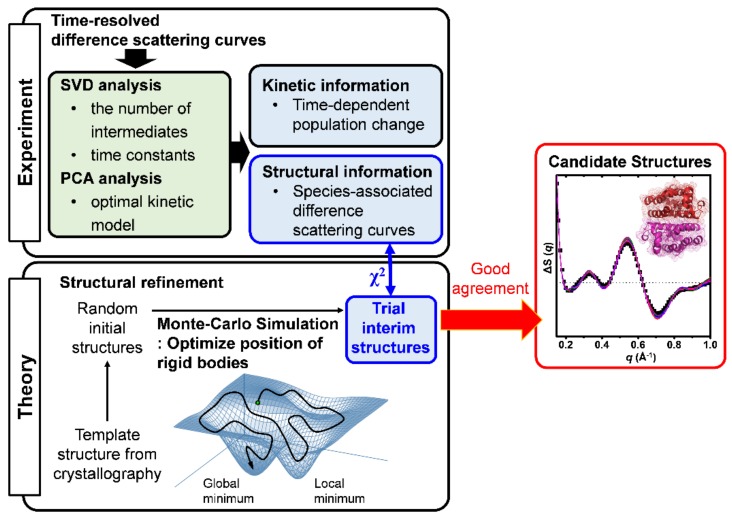
The schematic illustration of kinetic analysis and structure refinement. Time-resolved difference scattering curves of HbI were analyzed by SVD and PCA methods to obtain the information on the structural dynamics of HbI, including the time-dependent population changes and the species-associated difference scattering curves for the optimal kinetic model. The structural information of HbI was further analyzed by comparing the result of the structure refinement using a Monte-Carlo simulation. By moving rigid bodies randomly, initial structures were made from the template structure, which is the crystal structure related with the intermediate. The initial structures were optimized into the interim structures based on a Monte-Carlo simulation by minimizing the discrepancies (the χ^2^ value) between the curves of the theoretical structures and the species-associated difference scattering curve of the intermediate. The best structures, which have χ^2^ values less than the arbitrary criteria, were selected as the candidate structures corresponding to the intermediate.

To obtain detailed information on the structural dynamics of HbI, we first conducted a singular value decomposition (SVD) analysis, which is a method to separate the time-dependent information from the time-independent information, for the TRXSS data (Figure 2). As a result of the SVD analysis of the TRXSS data, the left singular vectors, the singular values, and the right singular vectors were obtained. The left singular vectors represent the time-independent difference scattering curves, which are basis vectors for reconstructing the species-associated difference curves for intermediates. The singular values represent the contributions of each singular vector. The right singular vectors contain the time-dependent information representing the temporal changes of the corresponding left singular vectors. The autocorrelation values of the left and right singular vectors were calculated to estimate the randomness of the vectors. Based on the singular values and the autocorrelation values of the corresponding singular vectors, we determined the minimal number of the significant singular vectors for describing the experimental TRXSS data, which are equivalent to the number of the distinguishable intermediates. The time constants of HbI kinetics were determined by fitting the significant right singular vectors simultaneously. Based on the SVD result, we performed a principal component analysis (PCA), which is a method for obtaining the species-associated information in the kinetic model (Figure 2). As a result of PCA, the species-associated difference X-ray scattering curves and the changes of the concentration corresponding to each intermediate in kinetic models were determined and then the time constants were optimized for the kinetic models again. We examined a variety of candidate kinetic models, which satisfy the numbers of the intermediates and of the time constants determined from the SVD result, by reducing the discrepancy between the experimental curves and the theoretical curves. As a result, we determined the best kinetic model with the smallest discrepancy.

**Figure 3:**
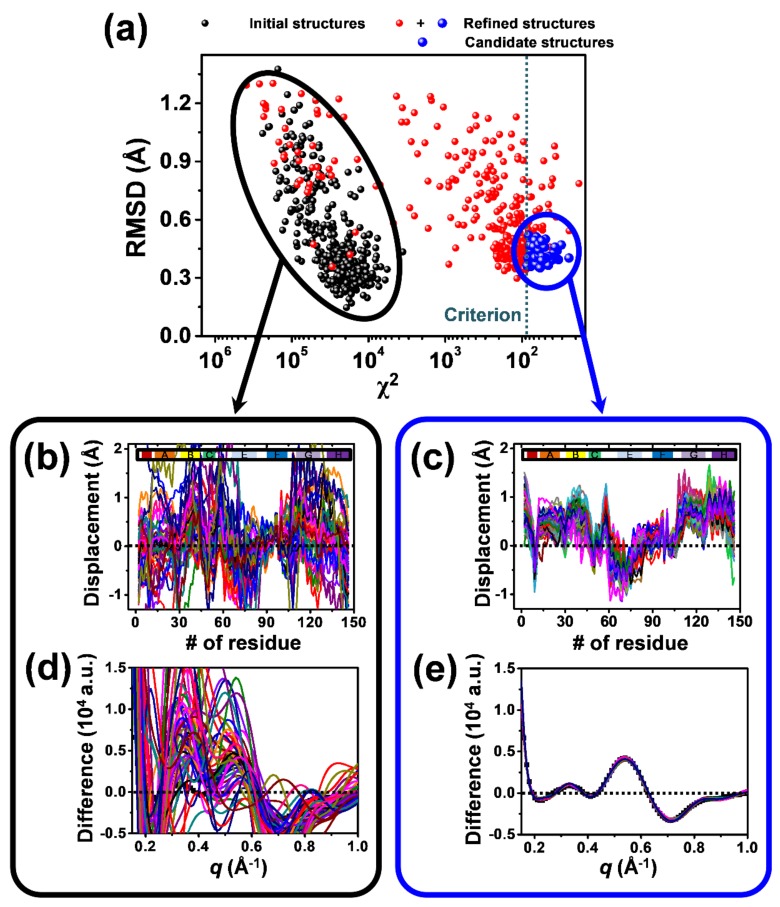
The structure refinement performed for the I_3_ intermediate of WT HbI (see Section 2.3) is shown as an example. Starting from 360 random initial structures generated by Monte Carlo simulations, we minimized the χ^2^ value, which is the degree of discrepancy between the experimental curve and the theoretical curve calculated from one of the starting structures, by exploring the structural space via Monte Carlo simulations guided by molecular dynamics (MD) force fields and simulated annealing. (**a**) The root mean square deviation (RMSD) for the initial structures (black dots) and the refined structures (red and blue dots) with respect to an arbitrary reference structure, which is the crystal structure of WT deoxy HbI (PDB ID: 4sdh) in this case, are plotted as functions of the χ^2^ values between the experimental curve and the theoretical curves. The best-refined structures (blue dots) are considered as the candidate structures. (**b**,**c**) Displacement plots for (**b**) 50 arbitrary structures chosen from among the 360 initial structures and (**c**) the 76 candidate structures. The displacement was calculated with respect to the crystal structure of WT HbI(CO)_2_ (PDB ID: 3sdh). Helices are indicated at the top of the plots. (**d**,**e**) Comparison of the experimental species-associated scattering curve with the theoretical scattering curves of (**d**) the 50 arbitrary structures and (**e**) the 76 candidate structures.

Although molecular structures cannot be directly calculated from X-ray solution scattering curves due to the random orientation of molecules in solution, the structural information contained in the solution scattering curves can be obtained by comparing them with the theoretical scattering curves of the structural simulation result. Therefore, we performed a structure refinement to obtain the candidate structures for the intermediates in the optimal kinetic model determined by PCA (Figure 2). In Figure 3, the structural refinement process of the I_3_ intermediate (see Section 2.3) for WT HbI is visualized as an example. As basic units for describing the structure of HbI, we set eighteen rigid bodies with helices and hemes. Based on a Monte Carlo simulation, the position and orientation of the rigid bodies were refined by minimizing the discrepancy (χ^2^ value) between the species-associated difference scattering curve and the theoretical curve corresponding to the refined structure (Figure 3a). We created different initial structures with randomly moved rigid bodies for each intermediate from the template structures (Figure 3b,d), which are the crystal structures corresponding to the intermediates and the deoxy state. All of the initial structures were refined individually. Among the refined structures, only the structures exhibiting a χ^2^ value below an arbitrary threshold were selected as the candidate structures for each intermediate (Figure 3c,e).

### 2.3. TRXSS Results of WT HbI

**Figure 4:**
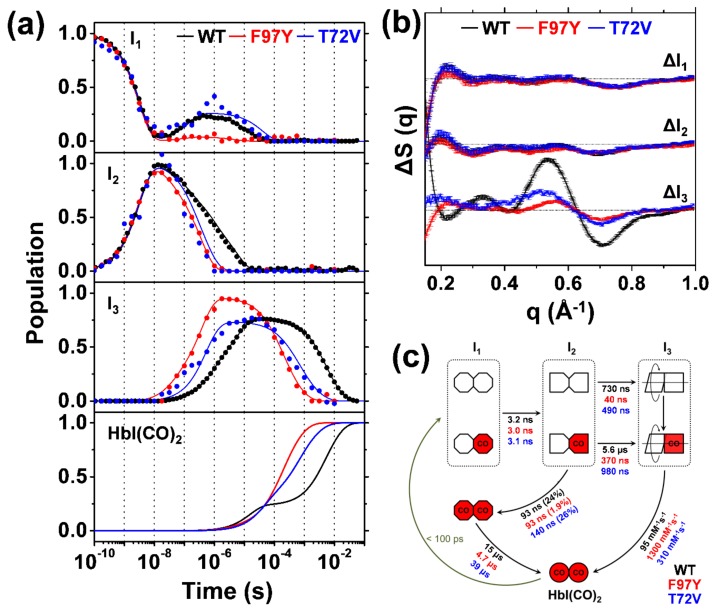
(**a**) The population changes of the three intermediates (I_1_, I_2_, and I_3_) and HbI(CO)_2_ for WT (black lines), F97Y (red lines), and T72V (blue lines) HbI obtained by kinetic analyses. The circles indicate the optimized populations obtained by fitting the experimental difference scattering curves with the species-associated difference scattering curves of the three intermediates shown in Figure 4b. (**b**) Species-associated difference scattering curves for the three intermediates of WT (black), F97Y (red), and T72V (blue) HbI. (**c**) A Kinetic model for HbI. The red (with “CO”) and white shapes represent ligated and photolyzed monomer subunits, respectively. The subunits of each intermediate are represented with different shapes, indicating the change in the tertiary structure during structural transitions. Two red octagons indicate a fully ligated form of I_1_, which is formed by the geminate recombination of CO with I_2_ and has an indistinguishable conformation compared to the photolyzed forms of I_1_. The two subunits of I_3_ rotating with respect to each other are represented to indicate the change in quaternary structure in the transition from I_2_ to I_3_. Time constants and bimolecular rate constants for WT, F97Y, and T72V HbI are shown in black, red, and blue, respectively. Percentage ratios for the geminate recombination of I_2_ to I_1_ are indicated in parenthesis.

The TRXSS difference curves of WT HbI [21] were measured and analyzed as described above. As a result of SVD and PCA, the species-associated difference scattering curves and the concentration changes for the three intermediates in the optimal kinetic model of WT HbI were determined (Figure 4). The optimal kinetic model of WT HbI is shown in Figure 4c. A 532 nm excitation of HbI(CO)_2_ leads to the formation of the I_1_ intermediate within 100 ps. With a time constant of 3.2 ns, I_1_ transits into the I_2_ intermediate. The I_2_ intermediate undergoes complex pathways, which are (i) a transition back to the I_1_ via a geminate recombination with a time constant of 93 ns and (ii) two transitions to the I_3_ intermediate with 730 ns and 5.6 μs time constants, respectively. The I_1_ intermediate generated by the geminate recombination from I_2_ (two red octagons in Figure 4c) decays back to the ground state with a time constant of 15.2 μs. The I_3_ intermediate recombines with CO in the solution with the bimolecular rate constant of 95 mM^−1^s^−1^. The biphasic transition from I_2_ to I_3_ suggests that two substates of I_2_ and I_3_ exist. The species-associated difference curves of the substates are the same, meaning that the substates have indistinguishable structures compared to each other. Since the branching ratio between transitions from I_2_ to I_3_ is dependent on the excitation fluence, the substates were assigned as fully and partially photolyzed forms (Figure 4c) [21]. The partially and fully photolyzed forms of HbI coexist in each intermediate but the structures of the two forms are indistinguishable in the TRXSS signal, indicating that the partially photolyzed form undergoes the same structural change as the fully photolyzed form effectively [21]. This observation provides direct experimental evidence that the photolysis of only a single subunit causes the same structural change as the photolysis of both subunits does, thereby revealing the allosteric effect in terms of structure. Besides, the close interaction between the subunits of HbI indicates that the HbI in the solution has the tertiary structural symmetry, which is consistent with studies using the molecular dynamics (MD) simulation [19] and resonance Raman spectroscopy [34].

**Figure 5:**
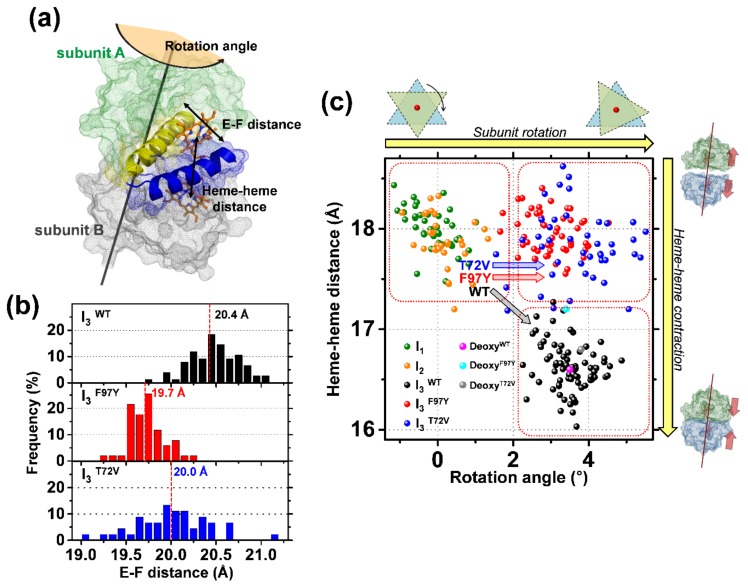
(**a**) The illustration of structural parameters inspected in candidate structures of various intermediates. E and F helices of the subunit A are represented in yellow and blue, respectively, and two hemes are shown in orange sticks. The rotation axis of the subunit rotation is shown with the black line. (**b**) The occurrence distribution of the E–F distances calculated for I_3_^WT^ (black), I_3_^F97Y^ (red), and I_3_^T72V^ (blue). (**c**) Heme–heme distances in the candidate structures of the intermediates plotted as a function of subunit rotation angle. The dots in green, orange, black, red, and blue corresponds to candidate structures of I_1_, I_2_, I_3_^WT^, I_3_^F97Y^, and I_3_^T72V^, respectively. The black, red, and blue arrows represent the I_2_ to I_3_ transition of WT, F97Y, and T72V HbI, respectively. Upon the I_2_ to I_3_ structural transition, WT HbI undergoes both subunit rotation and heme–heme distance contraction whereas T72V and F97Y HbI undergo only subunit rotation. For comparison, dots corresponding to crystal structures of deoxy forms of WT, F97Y, and T72V HbI (PDB IDs: 4sdh, 2aup, and 6hbi, respectively) compared with those of CO-bound forms (PDB IDs: 3sdh, 2auo, and 7hbi, respectively) are indicated in the magenta, cyan, and gray colors, respectively.

After obtaining the species-associated difference curves for the intermediates, the structural changes of each intermediate were examined by using the structure refinement aided by Monte-Carlo simulations. As shown in Table 2 and Figure 5, the heme–heme distance and the rotation angle between two subunits were determined. Compared to HbI(CO)_2_, the changes on the average heme–heme distance of I_1_ and I_2_ are –0.4 Å and –0.5 Å, respectively, and the average rotation angle between two monomers of I_1_ and I_2_ are only –0.1° and 0.1°, respectively, indicating that the I_1_ and I_2_ intermediates have R-like structures. In contrast of I_1_ and I_2_, the I_3_ intermediate shows the changes of 3.5° on the rotation angle and –1.8 Å on the heme–heme distance compared with HbI(CO)_2_, indicating that the I_3_ intermediate is more like the deoxy T state than the R state. In summary, the TRXSS data of WT HbI in solution revealed that WT HbI undergoes the I_2_-to-I_3_ transition (R-T transition) accompanying the subunit rotation and the contraction of the heme–heme distance.

### 2.4. TRXSS Results of F97Y HbI

TRXSS data of F97Y HbI were analyzed by using the same method applied for WT HbI [21]. While the same kinetic model of WT HbI works for F97Y HbI, the detailed structural dynamics of F97Y HbI is different from those of WT HbI as shown in Figure 4. The I_1_ intermediate of F97Y HbI decays with a time constant of 3 ns, which is similar to that of WT HbI (3.2 ns). The time constant for the geminate recombination from I_2_ of F97Y HbI was set to be the same with that of WT HbI (93 ns) since the contribution of the geminate recombination is too small (1.9%). The recovery from the geminately recombined I_1_ to HbI(CO)_2_ of F97Y HbI is accelerated more than three times (4.7 μs) compared with that of WT HbI (15 μs). The transitions from I_2_ to I_3_ of F97Y HbI are also greatly accelerated with 40 ns and 370 ns time constants for the fully and partially photolyzed forms, respectively. The bimolecular recombination of I_3_ of F97Y HbI (1300 mM^−1^s^−1^) is accelerated by almost thirteen times compared with that of WT HbI (95 mM^−1^s^−1^). The acceleration of the bimolecular recombination of F97Y HbI is consistent with the results of flash photolysis and equilibrium oxygen binding experiments [9,13,17,20,22].

The WT and F97Y HbI have essentially the same species-associated difference curves of I_1_ and I_2_ (Figure 4b), implying that the structures of I_1_ and I_2_ are the same for WT and F97Y within the signal-to-noise ratio of the current TRXSS. Unlike the I_1_ and I_2_, as shown in Figure 4b, the difference scattering curve corresponding to the I_3_ of F97Y HbI (I_3_^F97Y^) is significantly different from that of I_3_ of WT HbI (I_3_^WT^), indicating that the structure of I_3_^F97Y^ is different from that of I_3_^WT^. The average heme–heme distance of I_3_^F97Y^ is about 18.0 Å, which is virtually identical to that of the I_2_ (17.9 Å), whereas the average heme–heme distance of I_3_^WT^ is decreased by 1.3 Å compared with that of I_2_. On the other hand, the average rotation angle between the subunits of I_3_^F97Y^ is 3.0 ± 0.6°, which is close to that of I_3_^WT^ (3.5°). Therefore, the F97Y mutation affects the contraction of the heme–heme distance but not to the rotation of subunits. During the I_2_-I_3_ transition, the F97 residue of WT HbI is flipped into the subunit from the subunit interface, whereas the Y97 residue of the F97Y HbI stays in the interface between the subunits and therefore prevents the decrease of the heme–heme distance [17]. The relatively large heme–heme distance of I_3_^F97Y^ might contribute to the fast bimolecular recombination rate since the subunit interface of I_3_^F97Y^ is more accessible for CO ligands compared with that of I_3_^WT^. On the other hand, the average E–F distance in I_3_^F97Y^ is 19.7 ± 0.2 Å, which is smaller than that in I_3_^WT^ (20.4 Å) (Figure 5b). In a meta-analysis study [27], it was suggested that T-like states with low ligand affinity show larger E–F distances than R-like states with high ligand affinity. In this manner, the smaller E–F distance of I_3_^F97Y^ than that of I_3_^WT^ might be related with the acceleration of the bimolecular recombination rate.

### 2.5. TRXSS Results of T72V HbI

Similar to F97Y HbI, the TRXSS data of T72V HbI can be explained by the same kinetic model of WT HbI (Figure 4c) [24]. The I_1_ intermediate of T72V HbI is formed within 100 ps and undergoes the transition to the I_2_ intermediate with a 3.1 ns time constant, which is similar to those of WT and F97Y HbI. The geminate recombination of I_2_ occurs with a 140 ns time constant, whereas the transitions from I_2_ to I_3_ have time constants of 490 ns and 980 ns for the fully and partially photolyzed forms, respectively. The time constant for the geminate recombination of I_2_ of T72V HbI is slightly slower than those of WT and F97Y HbI, whereas the time constants of I_2_ to I_3_ transitions of T72V HbI are faster than those of WT but slower than those of F97Y HbI. The I_1_ population generated from I_2_ of T72V HbI recovers into HbI(CO)_2_ with a time constant of 39 μs, which is 2.6 times slower than that of WT HbI. Finally, the I_3_ intermediates of T72V HbI are bound with CO ligands with the second order rate constant of 310 mM^−1^s^−1^, which is almost three times faster than that of WT HbI. Similar to previous studies, the bimolecular recombination of T72V HbI is accelerated compared with that of WT HbI [9,13,17,20,22]. As shown in Figure 4c, the faster and slower time constants of I_2_-I_3_ transition can be assigned to the R-T transitions of fully photolyzed (τ_RT1_) and partially photolyzed (τ_RT2_) forms, respectively, based on the excitation fluence dependence of the branching ratio between two forms [21]. As the cooperativity between the subunits becomes higher, the difference of the time scales between the R-T transitions of fully and partially photolyzed forms will become smaller since the R-T transition of partially photolyzed form can be accelerated by the stronger interaction between the ligated and photolyzed subunits. Hence, we can predict the cooperativity of WT, F97Y, and T72V HbI by comparing the ratio between the two time constants of I_2_-I_3_ transitions of fully and partially photolyzed forms (τ_RT2_/ τ_RT1_). The τ_RT2_/ τ_RT1_ ratio decreases in the order of F97Y HbI (9.3) > WT HbI (7.7) > T72V HbI (2.0), which is in agreement with the trend of cooperativity of these HbI constructs obtained from the equilibrium oxygen binding experiments [13,17].

The I_1_ and I_2_ of T72V HbI have similar species-associated difference scattering curves compared with those of WT HbI (Figure 4b). Therefore, I_1_ and I_2_ of T72V HbI have the same structures as those of WT HbI. In contrast with I_1_ and I_2_, the I_3_ intermediate of T72V HbI (I_3_^T72V^) has a different structure compared with WT and F97Y HbI. I_3_^T72V^ exhibits the subunit rotation of 3.0 ± 0.6°, which is similar to those of I_3_^WT^ (3.5°) and I_3_^F97Y^ (3.7°), and the heme–heme distance of 17.8 ± 0.4 Å, which is clearly different from that of I_3_^WT^ (16.6 Å) but similar to that of I_3_^F97Y^ (18.0 Å). Unlike the I_3_^WT^, which shows a contraction of the heme–heme distance compared with I_2_ (17.9 Å), the I_3_^F97Y^ and I_3_^T72V^ have a similar heme–heme distance to I_2_. As in the case of I_3_^F97Y^, the larger heme–heme distance of I_3_^T72V^ than that of I_3_^WT^ might explain the faster bimolecular recombination of T72V than WT HbI. Overall, F97Y and T72V HbI undergo only the rotation of subunits, whereas WT HbI undergoes not only the subunit rotation but also the contraction of the heme–heme distance (Figure 5c). On the other hand, the E–F distance of I_3_^T72V^ (20.0 ± 0.4 Å) is smaller than that of I_3_^WT^ but larger than that of I_3_^F97Y^ (Figure 5b). In a meta-analysis of HbI structures, it was suggested that T-like states having low affinity possess larger E–F distances compared with R-like states having high ligand affinity, as mentioned above [27]. In line with the meta-analysis study, the ligand affinity of I_3_ decreases and the E–F distance of I_3_ increases in the order of F97Y, T72V, and WT HbI.

## 3. Discussion

**Figure 6:**
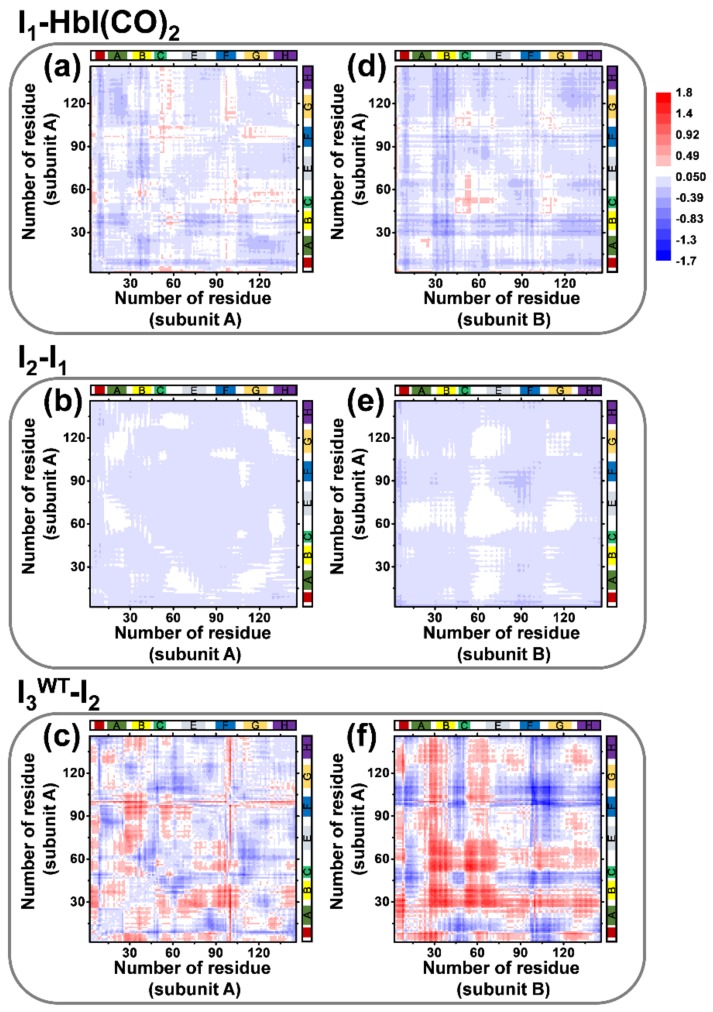
The averaged difference distance maps of the three intermediates of WT HbI. The difference distances between the intermediate and the reference structure were calculated. (**a**–**c**) The difference distance maps between subunit A of the intermediate and that of the reference structure. (**d**–**f**) The difference distance maps between subunit A of the intermediate and subunit B of the reference. The color scale was set to be the same for all maps for comparison and is shown on the right-top of the figure. Helices are labeled in the top and right of each panel.

To visualize the structural changes of WT HbI, difference distance maps of the candidate structures of individual intermediates were calculated and yielded averaged difference distance maps shown in Figure 6. Difference distance maps show the difference of the distance between any possible combination of Cα atoms in two difference structures. The averaged difference distance maps of I_1_, I_2_, and I_3_^WT^ shown in Figure 6 were calculated relative to HbI(CO)_2_, I_1_, and I_2_, respectively, to show structural changes involved in the structural transitions. The difference distances calculated for Cα atoms located in the same monomer subunit (subunit A) are shown in Figure 6a–c to represent the tertiary structural changes of WT HbI. The average difference distance map of subunit A in I_1_ relative to subunit A in HbI(CO)_2_ (Figure 6a) shows moderate tertiary structural changes. For example, the CD loop (residue 50−59) moves away from the heme (positive difference distance) and the E helix moves toward the heme (negative difference distance) in the transition from HbI(CO)_2_ to I_1_. The difference distance map of subunit A in I_2_ relative to that in I_1_ (Figure 6b) shows only a minor fluctuation, which indicates that the tertiary structure of I_2_ is very similar to that of I_1_. In the case of the difference distance map of subunit A in I_3_^WT^ compared with that in I_2_ (Figure 6c), it is shown that all helices in I_3_^WT^ undergo the tertiary structural rearrangement during the transition from I_2_ to I_3_^WT^. On the other hand, the difference distances calculated for Cα atoms located in different subunits are shown in Figure 6d–f to represent the quaternary structural changes of WT HbI. The difference distance map of the subunit A in I_3_^WT^ relative to the subunit B in I_2_ (Figure 6f) exhibits notable quaternary structural changes, whereas the difference distance maps between subunit A and B corresponding to the transitions from HbI(CO)_2_ to I_1_ (Figure 6d) and that from I_1_ to I_2_ (Figure 6e) show only a small amount of fluctuation. Clearly, the quaternary structural changes of WT HbI occur mainly during the transition from I_2_ to I_3_^WT^ as shown in the changes of the heme–heme distance and the rotational angle between subunits. In a study using NMR spectroscopy and MD simulation, the largest changes in the backbone NH dynamics that occur upon ligand binding were observed in the pre-A, B, F, and G helices in WT HbI [19]. Additionally, time-resolved crystallography studies [25,27] showed that the R-T transition upon ligand dissociation accompanies the structural changes in the E and F helices and the CD region. These results of the previous studies are consistent with the difference distance maps between I_3_^WT^ and I_2_ of the TRXSS result (Figure 6c,f).

The heme–heme distances in the crystal structures of deoxy states of WT, F97Y, and T72V HbI (16.6, 17.2, and 16.8 Å for WT, F97Y, and T72V HbI, respectively) are shorter than those of the CO-bound states (18.4 Å for WT, F97Y, and T72V HbI) (Figure 5c). In the result of TRXSS, I_3_^WT^, I_3_^F97Y^, and I_3_^T72V^, which correspond to the deoxy T states, have the heme–heme distances of 16.6, 18.0, and 17.9 Å, respectively. The heme–heme distances in I_3_^F97Y^ and I_3_^T72V^ are similar to that in I_2_ (17.9 Å), whereas I_3_^WT^ shows shorter heme–heme distance than I_2_. The heme–heme distances of I_3_^F97Y^ and I_3_^T72V^ are not contracted compared with that of I_2_, which is in contrast with the shortened heme–heme distances in the crystal phase of the deoxy states of F97Y and T72V HbI compared with those of the CO-bound states. To elucidate the discrepancy of the heme–heme distance contraction in the solution and the crystal phases, further studies are required. Meanwhile, an MD simulation study on HbI [31] demonstrated the role of interfacial water molecules in the conformational transition of HbI. The MD simulation study showed that the reduction of the number of the interfacial water molecules induces the conformational change from the deoxy-like to the oxy-like state, suggesting that the number of the interfacial water molecules and the structural changes, such as the ring flip of Phe97 and the change of heme–heme distance, are directly coupled. The numbers of the interfacial water molecules of I_3_^WT^, I_3_^F97Y^, and I_3_^T72V^ in TRXSS result are 17, 6, and 15, respectively (Table 2). The trends of the heme–heme distances determined from TRXSS are F97Y > T72V > WT in descending order, while the numbers of the interfacial water molecules for the intermediates were fixed as the values of the crystal structures, whose trends are F97Y > T72V > WT in the ascending order. The relation between the heme–heme distance and the number of the interfacial water molecules in the TRXSS result of HbI is consistent with the previous MD simulation study [31].

In a study on the SVD-aided pseudo-principal-component analysis, which is a method for obtaining model-dependent kinetic information directly from the experimental data without examining a variety of candidate models, we demonstrated that the TA data of WT HbI can be explained by using the similar kinetic framework of TRXSS results [30]. In the TA result, the biphasic transitions from I_2_ to I_3_ show the time constants of 190 ns and 1.1 μs, which are faster than those determined in the TRXSS results (730 ns and 5.6 μs). The faster transitions in the TA result than those in the TRXSS result were explained by the temporal delay between the local structural changes nearby the heme and the global structural changes of HbI. In other words, the local structural changes induced in the residues around the heme and the heme itself precedes the global structural changes of the protein matrix [68,83]. Therefore, the transition observed by TA, which is sensitive to the local structural changes of the chromophore and its nearby environment, is faster than the transition observed by TRXSS, which is sensitive to the global structural changes such as the change of the entire shape of molecules.

The bimolecular recombination between deoxy HbI and CO have been reported by using TA spectroscopy and equilibrium oxygen binding experiments [9,13,17,20,22]. The bimolecular recombination rates of CO for the deoxy state were reported as 90 [9,17], 4300–4400 [17,20], and 900–1800 mM^−1^s^−1^ [13,22] for WT, F97Y, and T72V HbI, respectively. For studies reported two rates for the recombination, slower rate constants are selected for the following discussion, since the slow recombination corresponds to the recombination of the low-affinity T state [22]. While the trends of the rates of mutants in TRXSS result are the same as F97Y > T72V > WT in descending order compared to previous studies, the bimolecular recombination rates determined by TRXSS for F97Y and T72V HbI are slower (1300 and 310 mM^−1^s^−1^, respectively) than those reported by the previous studies, whereas that for WT HbI is similar as 90 mM^−1^s^−1^ (Figure 4c). The difference of the bimolecular recombination between TRXSS and the previous studies might originate from the difference of the experimental conditions such as the concentration of HbI. An alternative possibility is the different kinetic models for describing the experimental data. The rate constant of bimolecular recombination for the TRXSS results of HbI was numerically determined by using the kinetic model shown in Figure 4c. On the contrary, in the previous studies reporting the bimolecular recombination rate constants [9,13,17,22], the kinetic equivalent of a two-state model (or MWC (Monod, Wyman, and Changeux) model [84]) was used to describe the HbI kinetics. The two-state model assumes equilibrium of the R and T states and includes the kinetic parameters of the allosteric constant (*c*), the equilibrium constant between two states (*L*), bimolecular recombination rate constants, and ligand dissociation rate constants [29].

In a study of WT HbI using transient grating (TG) spectroscopy [36], which is a time-resolved experiment measuring refractive index changes of the sample that can detect the diffusion coefficients of the intermediates and reactant, we explained the TG results by adopting a sequential kinetic model. The kinetic parameters determined by the fitting of TG signals of WT HbI are three reaction rates, two diffusion coefficients corresponding to HbI and CO, and a thermal diffusion coefficient. Based on the sequential kinetic model, the kinetics of HbI from the TG signal were described as follows. First, the R state of HbI undergoes the tertiary structural change into the tertiary T state with a 1.4 ± 0.4 μs time constant after the photodissociation of CO ligands, and then the quaternary R-T transition occurs with an 8.7 ± 4.3 μs time constant. After the transitions are completed, the bimolecular recombination between HbI and CO occurs with a 5.3 ms pseudo-first-order time constant. However, this sequential kinetic model of the R-T transition used for the TG result is contrary to the biphasic model for the I_2_-I_3_ transition used for the TRXSS results. Fortunately, since the sequential model and the biphasic model in these cases can be reduced into the simple exponential series, the application of the biphasic model for the R-T transition to the TG signals is possible without any changes on the time constants. If we introduce the biphasic model to the TG result, two 1.4 μs and 8.7 μs time constants correspond to two R-T transition processes of the fully and partially photolyzed HbI, respectively. These time constants determined from the TG result are slower than those of the TRXSS result (730 ns and 5.6 μs) (Figure 4c) [21] and those of the TA result (190 ns and 1.1 μs) [30]. The time constants of R-T transition determined by the TG result are closer to the TRXSS result than the TA result. Since the TG experiment detects the spectrally silent characteristics such as volume change and the changes in diffusion coefficients, TG signals can reflect the global structural changes of protein. In this regard, the time constants for the R-T transition of HbI determined by the TG result are slower than those determined by the TA result because of the temporal delay between the global and local conformational changes as the discrepancy between the TRXSS and TA results is explained above.

## 4. Conclusions

In this review, we summarized the TRXSS results of WT, F97Y, and T72V HbI [21,24]. By taking advantage of the structure-sensitive TRXSS experiment and the strategic structure refinement, the structural dynamics of the HbI constructs was elucidated. In particular, the biphasic transition from I_2_ to I_3_ is necessary to explain the TRXSS result and the fully and partially photolyzed forms share an indistinguishable structure, indicating that the partially photolyzed form exhibits the same structural change as the fully photolyzed form, which is a direct evidence for the allosteric structural transition. Additionally, it was revealed that the contraction of the heme–heme distance is absent in F97Y and T72V HbI during the R-T transition, whereas the rotation of two subunits is exhibited for all of WT, F97Y, and T72V HbI. The preservation of the heme–heme distance in F97Y and T72V HbI could be responsible for the acceleration of the bimolecular recombination between the HbI and CO ligands. However, the rate of the bimolecular recombination does not depend on the heme–heme distance alone, as that of F97Y is 4.2 times larger than that of T72V HbI. We suggest that the distance between the E–F helices might be related to the trends of the changes of the bimolecular recombination rate on the basis of a meta-analysis study [27].

## Figures and Tables

**Table 1:** The number of interfacial water molecules in the crystal structures of WT, F97Y, and T72V HbI.

	WT HbI	F97Y HbI	T72V HbI
HbI(CO)_2_	11 ^a^	9 ^c^	11 ^e^
Deoxy HbI	17 ^b^	6 ^d^	15 ^f^

The number of the interfacial water molecules were reported in X-ray crystallography studies of WT HbI [12], F97Y HbI [17], and T72V HbI [23]. PDB IDs of the crystal structures are ^a^ 3sdh, ^b^ 4sdh, ^c^ 2auo, ^d^ 2aup, ^e^ 7hbi, and ^f^ 6hbi.

**Table 2:** The RMSD values for the whole protein, iron−iron distances between the two hemes, numbers of interfacial water molecules, and subunit rotation angles for the averaged refined structures of the intermediates (I_1_, I_2_, and I_3_) and the deoxy-HbI crystal structure (PDB ID: 4sdh) with respect to the HbI(CO)_2_ crystal structure (PDB ID: 3sdh) ^a^.

	RMSD (Å)	Iron−Iron Distance (Å)	Number of Interface Water Molecules	Rotation Angle (deg)
HbI(CO)_2_ (3sdh)	−	18.4	11	−
deoxy-HbI (4sdh)	0.6	16.6	17	3.5
I_1_	0.4 (±0.05)	18.0 (±0.2)	9 (fixed) ^b^	−0.1 (±0.5)
I_2_	0.4 (±0.06)	17.9 (±0.3)	9 (fixed) ^b^	0.1 (±0.5)
I_3_^WT^	0.7 (±0.05)	16.6 (±0.2)	17 (fixed) ^b^	3.5 (±0.6)
I_3_^F97Y^	0.8 (±0.04)	18.0 (±0.2)	6 (fixed) ^b^	3.0 (±0.6)
I_3_^T72V^	0.8 (±0.06)	17.8 (±0.4)	15 (fixed) ^b^	3.7 (±1.0)

−: The RMSD and the subunit rotation angles were determined with respect to the structure of HbI(CO)_2_. ^a^ Values in parentheses are standard deviations among the candidate structures. ^b^ The numbers of interfacial water molecules for the intermediates were fixed as the values of the crystal structures, which are the crystal structure of the M37V mutant HbI formed at 5 ns after the photolysis of CO ligands (PDB ID: 2grz) [25] for I_1_ and I_2_, the deoxy crystal structure of WT HbI (PDB ID: 4sdh) [12] for I_3_ of WT HbI (I_3_^WT^), the deoxy crystal structure of F97Y HbI (PDB ID: 2aup) [17] for I_3_ of F97Y HbI (I_3_^F97Y^), and the deoxy crystal structure of T72V HbI (PDB ID: 6hbi) [23] for I_3_ of T72V HbI (I_3_^T72V^).

## Abbreviations

Hb	Human hemoglobin
HbI	Homodimeric hemoglobin from *Scapharca inaequivalvis*
R state	Relaxed state
T state	Tense state
WT	Wild-type
T72	Thr72
F97	Phe97
TRXSS	Time-resolved X-ray solution scattering
TRXL	time-resolved X-ray liquidography
XFEL	X-ray free-electron laser
RMSD	Root-mean square deviation
TA	Transient absorption
SVD	Singular value decomposition
PCA	Principle component analysis
F97Y HbI	HbI mutant construct of Phe97 to Tyr
T72V HbI	HbI mutant construct of Thr72 to Val
I_3_^WT^	I_3_ intermediate for wild-type HbI
I_3_^F97Y^	I_3_ intermediate for F97Y HbI
I_3_^WT^	I_3_ intermediate for T72V HbI
cryo-EM	Cryo-electron microscopy
NMR	Nuclear magnetic resonance
MD	Molecular dynamics
TG	Transient grating
HbI(CO)_2_	HbI ligated with CO ligands
